# Crystallographic analysis of engineered polymerases synthesizing phosphonomethylthreosyl nucleic acid

**DOI:** 10.1093/nar/gkac792

**Published:** 2022-09-17

**Authors:** Mohammad Hajjar, Nicholas Chim, Chao Liu, Piet Herdewijn, John C Chaput

**Affiliations:** Department of Pharmaceutical Sciences, University of California, Irvine, CA 92697-3958, USA; Department of Pharmaceutical Sciences, University of California, Irvine, CA 92697-3958, USA; Medicinal Chemistry, Rega Institute for Medical Research, KU Leuven, Herestraat 49, 3000 Leuven, Belgium; Medicinal Chemistry, Rega Institute for Medical Research, KU Leuven, Herestraat 49, 3000 Leuven, Belgium; Department of Pharmaceutical Sciences, University of California, Irvine, CA 92697-3958, USA; Department of Chemistry, University of California, Irvine, CA 92697-3958, USA; Department of Molecular Biology and Biochemistry, University of California, Irvine, CA 92697-3958, USA; Department of Chemical and Biomolecular Engineering, University of California, Irvine, CA 92697-3958, USA

## Abstract

Xeno-nucleic acids (XNAs) are synthetic genetic polymers with backbone structures composed of non-ribose or non-deoxyribose sugars. Phosphonomethylthreosyl nucleic acid (pTNA), a type of XNA that does not base pair with DNA or RNA, has been suggested as a possible genetic material for storing synthetic biology information in cells. A critical step in this process is the synthesis of XNA episomes using laboratory-evolved polymerases to copy DNA information into XNA. Here, we investigate the polymerase recognition of pTNA nucleotides using X-ray crystallography to capture the post-catalytic complex of engineered polymerases following the sequential addition of two pTNA nucleotides onto the 3′-end of a DNA primer. High-resolution crystal structures reveal that the polymerase mediates Watson–Crick base pairing between the extended pTNA adducts and the DNA template. Comparative analysis studies demonstrate that the sugar conformation and backbone position of pTNA are structurally more similar to threose nucleic acid than DNA even though pTNA and DNA share the same six-atom backbone repeat length. Collectively, these findings provide new insight into the structural determinants that guide the enzymatic synthesis of an orthogonal genetic polymer, and may lead to the discovery of new variants that function with enhanced activity.

## Graphical Abstract

**Graphical Abstract:**
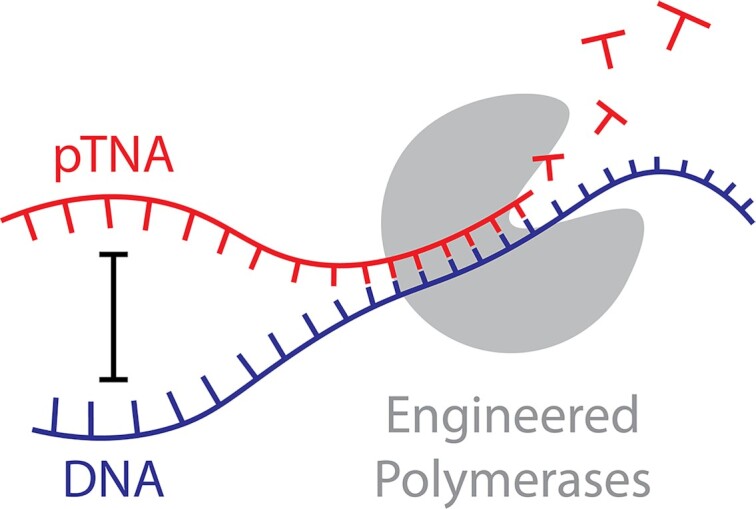
Structural insights into the enzymatic synthesis of an orthogonal genetic polymer that does not form antiparallel duplex structures with DNA and RNA.

## INTRODUCTION

All organisms store their genetic information in DNA and propagate that information through a process of genome replication and cell division. In a test tube, this process of information storage and propagation has been extended to xeno-nucleic acids (XNAs), artificial genetic polymers in which the natural deoxyribose sugar found in DNA has been replaced with an alternative sugar or sugar-like moiety (1). In a typical XNA replication cycle, XNA templates are reverse transcribed into DNA, amplified using the polymerase chain reaction (PCR) and transcribed back into XNA (2). This process of copying information back and forth between DNA and XNA requires engineered polymerases that are carefully evolved in the laboratory to recognize specific XNA substrates (3). Although XNA replication systems have been used to evolve nucleic acid aptamers and catalysts with backbone structures composed entirely of XNA (4–13), others have contemplated the potential for XNA to serve as a repository for synthetic biology information that is maintained inside a cell (14). Such XNA-modified organisms could overcome the biocontainment problem facing current genetically modified organisms by providing a genetic firewall to separate synthetic biology information from the natural DNA information of the cell (15). XNA-modified organisms that escape containment would lose their synthetic biology information by being deprived of critical XNA building block materials that are not provided by nature but required to maintain a replicating XNA chromosome (16).

**Figure 1.**
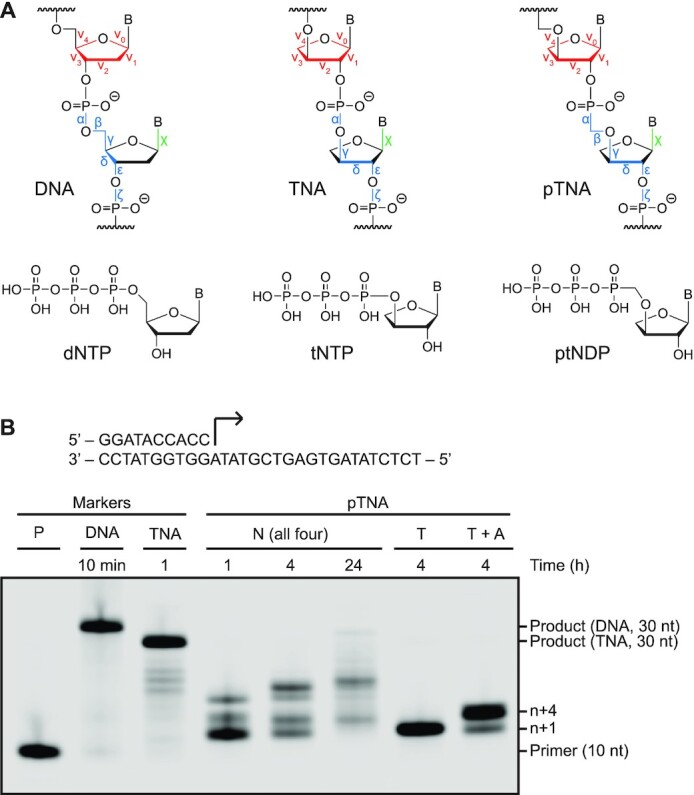
pTNA synthesis by Kod-RI TNA polymerase. (**A**) Molecular structures of the linearized backbones of DNA, TNA and pTNA and their nucleotides. Backbone, sugar and glycosidic torsion angles are colored blue, red and green, respectively. (**B**) Kod-RI-mediated extension of a 5′ IR800-labeled DNA primer annealed to a DNA template with ptNDP substrates. Full-length DNA and TNA products along with the starting primer are provided as markers. The reaction contained 0.5 μM IR800-labeled primer–template duplex, 100 μM of the specified nucleotides and 0.5 μM Kod-RI (pre-primed with 1 mM MnCl_2_ for TNA and pTNA), in 1× ThemoPol^®^ buffer for the specified time at 55°C.

Phosphonomethylthreosyl nucleic acid (pTNA, also abbreviated PMT and tPhoNA; Figure 1A) is considered a possible XNA candidate for information storage in cellular systems (17). pTNA is an unusual example of an XNA; it contains modifications in both the sugar and phosphodiester moieties with α-l-threofuranosyl sugars connected by 3′→2′ phosphonomethyl backbone linkages. This chemical configuration makes pTNA stable to nucleases that degrade natural genetic polymers but prevents pTNA from forming stable antiparallel duplex structures with complementary strands of DNA and RNA (17). As such, pTNA is viewed as an orthogonal genetic polymer, as it does not cross-pair with the genetic polymers of life and, therefore, would not interfere with the normal nucleic acid processes of the cell.

Efforts to advance pTNA as an orthogonal genetic polymer have focused on developing engineered polymerases that can faithfully copy DNA sequences into pTNA. The most recent example is TgoT-EPFLH, an engineered polymerase that derives from a replicative B-family DNA polymerase isolated from the archaeal hyperthermophilic species *Thermococcus gorgonarius* (Tgo) (17). In the presence of mutagenic manganese ions, TgoT-EPFLH has been shown to synthesize pTNA oligonucleotides of lengths up to 57 pTNA residues. This finding, as well as related work on glycerol nucleic acid (GNA) (18), demonstrates that stable Watson–Crick base pairing between the primer and template strands is not an essential criterion for the enzymatic synthesis of XNA polymers on DNA templates.

Here, we investigate the structural determinants that enable engineered polymerases to synthesize short segments of an orthogonal genetic polymer on a DNA template. Since we were unable to crystallize Tgo-EPFLH, we focused our analysis on Kod-RI, a laboratory-evolved polymerase that was previously developed to synthesize α-l-threose nucleic acid (TNA), a close structural analogue of pTNA (19). X-ray crystal structures of the post-catalytic complex showing Kod-RI bound to a DNA substrate that had been extended with either two pTNA adenosine residues (ptA) or two 2′-deoxyadenosine residues (dA) were solved to high resolution. Both structures were compared to an identical structure previously solved by extending Kod-RI with two TNA adenosine residues (tA) (20). Additionally, we solved the crystal structure for the post-catalytic complex of Kod-RSGA, a further evolved TNA polymerase relative to Kod-RI (21), bound to the same primer–template duplex following extension with two pTNA residues. Close inspection of the active site region of each polymerase reveals features that are unique to each system. Together, this work expands our understanding of the mechanisms that natural and engineered polymerases utilize to recognize their cognate substrates. We postulate that this information could be used to create new variants with improved XNA synthesis activity.

## MATERIALS AND METHODS

### Oligonucleotides and nucleotides

DNA oligonucleotides were purchased from Integrated DNA Technologies (IDT) (Supplementary Table S1). Primers used for polymerase extension assays were purchased as a 5′-hexynyl derivative, and then labeled with an IR fluorescent dye via click chemistry using a CuBr solution and IR Dye Azide 680 or 800 CW (Glen Research). The template used for X-ray crystallography was 5′-Cy5 labeled and HPLC purified by IDT. TNA and pTNA nucleotides were chemically synthesized as previously described (17,22,23).

### Protein expression and purification

Kod-RI and Kod-RSGA were cloned into pET21a (Novagen) and expressed and purified from *Escherichia coli* as previously described (20). Briefly, the gene for each polymerase (Kod-RI or Kod-RSGA) was PCR amplified from a pGDR11 expression vector using primers P_f_ and P_r_ containing NdeI and NotI restriction enzyme sites, respectively. Digestion using NdeI and NotI restriction enzymes (New England Biolabs) and ligation introduced the amplicons into pET21a (Novagen). The new plasmids were sequence verified (Retrogen) prior to use. Then, each plasmid was transfected into Acella^®^ cells (Edge BioSystems). The cells were grown aerobically at 37°C in LB media containing 100 μg ml^−1^ ampicillin to an OD_600_ of 0.8 and induced with 1 mM isopropyl β-d-thiogalactoside (Gold Biotechnology) at 18°C for 20 h. Cells were harvested by centrifugation (3315 × *g*, 20 min, 4°C).

Harvested cells were lysed in lysis buffer (Supplementary Table S2) by sonication, and the lysate was centrifuged (23 708 × *g*, 30 min, 4°C). The clear supernatant was heat treated (70°C, 20 min) to precipitate endogenous *E. coli* proteins by centrifugation (23 708 × *g*, 30 min, 4°C). The supernatant was filtered through a 0.2-μm sterilized filter, and then loaded onto 5-ml HiTrap Q HP in tandem with a 5-ml heparin HP column (GE Healthcare) and washed with low-salt buffer. The Q column was removed before eluting the polymerase in a high-salt buffer (Supplementary Table S2) with a linear gradient. Individual fractions were visualized by SDS-PAGE, pooled and concentrated using a 30-kDa cutoff Amicon centrifugal filter (Millipore). The concentrated protein was purified by size exclusion chromatography (Superdex 200 HiLoad 16/600, GE Healthcare) pre-equilibrated with Kod buffer (Supplementary Table S2) and concentrated to 10 mg ml^−1^.

### Primer extension assays

Primer extension assays were performed in 20 μl reaction volumes containing 1× ThermoPol^®^ reaction buffer (New England Biolabs; Supplementary Table S2), 0.5 μM IR-labeled primer (P_1_) and 0.5 μM template (T_1_) (Supplementary Table S1). The primer and template strands were annealed by incubating at 95°C for 5 min and cooling on ice for 5 min, and the duplex was combined with 100 μM of the appropriate nucleotides. The reactions were initiated by adding 0.5 μM engineered polymerase pre-incubated with 1 mM MnCl_2_ and incubated at 55°C for the specified times. DNA full-length markers were extended by Kod-RI or Kod-RSGA without MnCl_2_. The reactions were quenched with stop buffer (Supplementary Table S2), denatured for 10 min at 95°C and analyzed by 15% denaturing polyacrylamide gel electrophoresis (10 W, 1.5 h). Gels were imaged using a LI-COR Odyssey CLx imager.

### Protein crystallization

Laboratory-evolved Kod polymerases were crystallized with the identical DNA primer–template duplex following the addition of two adenosine residues for pTNA or DNA. We refer to these complexes as Kod-RI/pTNA, Kod-RI/DNA and Kod-RSGA/pTNA. For each complex, equal amounts of the primer P_c_ and the Cy5-labeled template T_c_ (Supplementary Table S1) were annealed in Kod buffer (Supplementary Table S2) supplemented with 20 mM MgCl_2_ (Honeywell, Fluka) by heating to 95°C for 5 min and cooling for 10 min at 10°C. Next, 1.5 molar equivalents of the duplex were incubated with 5 mg ml^−1^ polymerase at 37°C for 30 min to form the binary complex in solution. Finally, 5 molar equivalents of the appropriate nucleotide (dATP or ptADP) were incubated with the binary complexes at 37°C for 30 min to form the post-catalytic complex used for protein crystallization.

Optimal protein crystallization conditions were identified by screening commercial protein crystallization kits (Hampton Research and Qiagen) using a pipetting robot (Mosquito, TTP Techlab Ltd) to set up 0.5 μl hanging drops of equal volumes of sample and crystallization solutions in 96-well format containing 100 μl of crystallization solutions. Successful conditions were optimized over pH and precipitant concentration ranges using 24-well trays with 2 μl drops and 500 μl crystallization solutions. Crystallization reagents were purchased from commercial suppliers and were of analytical grade. Stock solutions of sodium acetate (BioWorld) and sodium citrate tribasic dihydrate (BioXtra, Sigma-Aldrich) were filtered before use. The crystallization condition of the reported Kod-RI/pTNA and Kod-RI/DNA structures was 0.2 M sodium acetate, 0.1 M sodium citrate (pH 4.5–5.5) and 7–10% (w/v) polyethylene glycol 4000, and the crystallization solution for the reported Kod-RSGA/pTNA structure was 0.1 M Mg(OAc)_2_ tetrahydrate, 0.1 M MOPS (pH 7) and 7–10% polyethylene glycol 8000. Crystal trays were stored in the dark at 24°C, and crystals grew in ∼4 weeks. Crystals of the reported structures were cryoprotected and dehydrated in 25% ethylene glycol and preserved in liquid nitrogen before data collection at the synchrotron.

### Data collection, processing and structural determination

The Kod-RI/pTNA dataset was collected at the Advanced Light Source (ALS) at the Lawrence Berkeley National Laboratory on 21 May 2021 at 100 K using a single wavelength protocol (1.0 Å) on beamline 8.2.1 equipped with a CCD detector, ADSC Quantum 315r. The dataset was reduced, integrated and scaled by XDS (24). Pointless (25) and aimless (26) were run for further space group determination and final data merging. Molecular replacement was performed using Phaser (27) using the previously published Kod-RI/TNA model (PDB ID: 5VU9) (20). The two TNA nucleotides were replaced by pTNA ligands created using eLBOW (28). Iterative rounds of manual building using Coot (29) followed by refinement using phenix.refine (30) were performed in Phenix 1.13-2998 (31). Residue 757 until the C-terminus of the 774-residue Kod-RI and the template’s 5′ terminal nucleotide were not built due to poor electron density. Final rounds of refinement employed TLS parameters, where Kod-RI was partitioned into five TLS groups (residues 1–110, 111–214, 215–337, 338–552 and 553–756). Two additional TLS groups were the template and the primer. The stereochemistry and geometry of all structures were validated with MolProbity (32), and the final refinement parameters are summarized in Supplementary Table S3. Final coordinates and structure factors have been deposited into the Protein Data Bank (PDB ID: 7RSR). Polder maps (33) were created using the collected dataset and the final refinement of the model.

The Kod-RI/DNA dataset was collected at the Stanford Synchrotron Radiation Lightsource at the National Accelerator Laboratory on 21 July 2021 at 100 K using a single wavelength protocol (1.0 Å) on beamline BL 12-1 using a pixel detector, Dectris Eiger X 16M. The indexing, integration and merging were done using iMOSFLM (34), and molecular replacement was performed using Phaser on an early refinement of the Kod-RI/pTNA model. The same refinement strategy was employed, and the same residues were built. The TLS groups were residues 1–225, 226–304, 305–552 and 553–765, template and primer. The structure was deposited with PDB ID: 7RSS.

The Kod-RSGA/pTNA dataset was collected at ALS on 20 December 2021 at 100 K using a single wavelength protocol (1.0 Å) on beamline 5.0.1 equipped with a pixel detector, Dectris PILATUS3 2M. The dataset was processed using XDS, and molecular replacement was performed using Phaser on the Kod-RI/pTNA model and followed the same refinement strategy. The TLS groups were residues 1–110, 111–214, 215–337, 338–552 and 553–756, template and primer. The structure was deposited with PDB ID: 7TQW.

### Structural measurements, calculations and visualization

Endocyclic torsion angles were extracted using PROSIT (35). Exocyclic torsion angles, base-pair parameters and base-pair step parameters were extracted using Web 3DNA (36). Torsion angles for unnatural moieties not recognized by Web 3DNA and phosphate to phosphate (or phosphonate) distances were measured in PyMOL by Schrödinger (https://pymol.org/2/). Protein RMSD measurements were based on the alpha carbons and calculated in PyMOL. All structural alignments and molecular graphics were prepared using PyMOL; alignments in Figure 3 were based on the six nucleotides displayed for each genetic system (T4, T5, C6, G11, A12 and A13).

## RESULTS

### pTNA synthesis by Kod-RI TNA polymerase

Kod-RI is an engineered TNA polymerase that was evolved from a replicative B-family DNA polymerase isolated from the archaeal hyperthermophilic species *Thermococcus kodakarensis* (Kod) (19). The polymerase harbors the A485R and E664I mutations required for TNA synthesis on DNA templates, as well as the 3′,5′-exonuclease silencing mutations (D141A, E143A) that prevent the removal of newly added TNA residues. The ability for Kod-RI to synthesize TNA has been extensively studied by X-ray crystallography and structures now exist for each of the major intermediates in the TNA synthesis pathway, including the apo, binary, open and closed ternary complexes of the pre-catalytic state as well as the translocated post-catalytic product (20). Recognizing that pTNA is a close structural analogue of TNA (Figure 1A), we wondered whether Kod-RI might also facilitate pTNA synthesis by recognizing chemically prepared ptNDP nucleotides (17,37), the diphosphate monophosphonate building blocks of pTNA, in a template-dependent primer extension reaction (Figure 1A). Our findings indicate that Kod-RI is capable of synthesizing trace amounts of full-length pTNA after 24 h of incubation at 55°C (Figure 1B). Although inadequate for most synthetic biology applications (38), previous studies have shown that this level of activity can be improved by directed evolution using polymerase engineering techniques that have been established to evolve DNA polymerases for the ability to transcribe and reverse transcribe other types of XNA polymers (3).

### Crystal structure capturing Kod-RI incorporating pTNA

**Figure 2.**
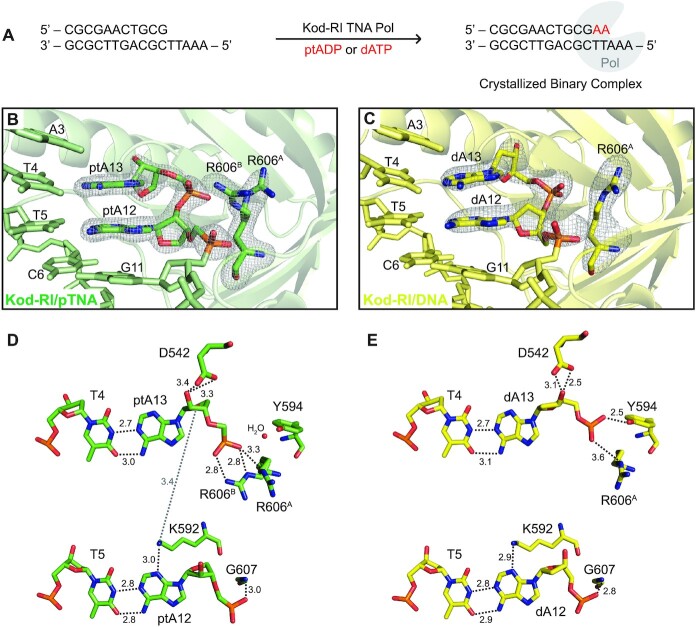
Active site view of Kod-RI TNA polymerase showing pTNA incorporation. (**A**) Schematic representation of the crystallized binary complex following the addition and translocation of two adenosine nucleotides. (**B**) View of the active site of Kod-RI incorporating pTNA with a 2F_o_–F_c_ composite polder map contoured at 7.5*σ* for the incorporated ptA12 and ptA13, and another polder map contoured at 5.0*σ* for both conformations of arginine 606, labeled A and B. The exonuclease domain is hidden to avoid obstruction of the view. (**C**) Kod-RI incorporating DNA with polder maps contoured at 7*σ* for DNA (dA12 and dA13) and 4*σ* for the single conformation of arginine 606. (**D**, **E**) The intermolecular interactions observed in the active site with distances reported in angstroms. Color scheme: structure obtained for the pTNA extended primer (green) and DNA extended primer (yellow).

Kod-RI is a large (90 kDa), highly dynamic engineered polymerase with a disk-shaped architecture that encompasses the N-terminal, exonuclease and catalytic domains of the protein. Similar to other DNA polymerases, the catalytic domain resembles a right hand consisting of the thumb, fingers and palm subdomains (Supplementary Figure S1A). To allow direct comparison to our previously solved X-ray crystal structure of the post-catalytic complex of Kod-RI bound to a DNA duplex that had been extended by two TNA adenosine residues (tA) (20), crystals of Kod-RI capturing the extended product of pTNA synthesis were grown from primer extension reactions that were performed in buffered solutions (Figure 2A). A parallel set of reactions performed with 2′-deoxyadenosine triphosphate (dATP) allowed us to obtain the DNA extended product as a control for understanding how Kod-RI recognizes the natural all-DNA duplex. Crystals obtained from both experiments resolved to resolutions of 1.98 and 2.71 Å for pTNA and DNA, respectively, and the structures were solved by molecular replacement (Supplementary Table S3) using coordinates from our previous Kod-RI/TNA structure (PDB ID: 5VU9). Both of the new Kod-RI structures are highly homologous to the original Kod-RI/TNA polymerase structure with calculated Cα RMSD values of 0.567 and 0.578 Å for the Kod-RI/pTNA and Kod-RI/DNA complexes, respectively. 2F_o_–F_c_ polder maps contoured at 7.5*σ* and 7.0*σ* for pTNA and DNA, respectively, unambiguously confirm the presence of the ptA residues at positions 12 and 13 of the primer strand as well as the equivalent dA nucleotides on the control all-DNA structure (Figure 2B and C).

The nucleotide addition products reside in the enzyme active site of Kod-RI as canonical Watson–Crick base pairs with their complementary thymine bases on the DNA template. Close inspection of the newly added residues failed to identify any differences in the hydrogen bonding pattern of the Watson–Crick base pair even though pTNA does not form a stable duplex with DNA in solution. In fact, heavy atom distances (2.7–3.0 Å) observed for the ptA–dT base pairs at positions 12 and 13 of the primer strand are nearly identical to the hydrogen bonding distances observed for the tA–dT and dA–dT base pairs in the equivalent Kod-RI structures produced by extending the primer with either tATP or dATP, respectively (Figure 2D and E, and Supplementary Figure S2). No signs of duplex destabilization were observed in the pTNA structure, indicating that Kod-RI likely forces pTNA–DNA hybridization by acting as a large molecular clamp to hold the complementary pTNA and DNA strands together.

To better understand the molecular interactions responsible for pTNA synthesis, we examined the amino acid residues in the polymerase active site that directly contact the pTNA nucleotides. In the Kod-RI structure of the pTNA extended primer, we observe a novel alternative conformation for R606 (labeled R606^B^) that is not present in the Kod-RI structures solved for the DNA or TNA extended primers (Figure 2 and Supplementary Figure S2A). Located on β26 of the thumb subdomain (Supplementary Figure S1B), the positively charged guanidino group of R606^B^ forms a strong ion pair interaction with the negatively charged phosphonate group connecting the newly added ptA12 and ptA13 nucleotides (Figure 2D). In the Kod-RI structure of the DNA extended primer, the analogous phosphate group forms a hydrogen bond to Y594 on β25 of the thumb subdomain (Figure 2E). Interestingly, neither of these interactions are observed in the previously solved structure of Kod-RI that was extended with TNA (Supplementary Figure S2), which could be due to the fact that TNA has a backbone repeat unit that is one atom shorter than that of pTNA or natural DNA.

Other structural differences observed in the polymerase active site include a bridging water interaction between Y594 and the phosphate group linking ptA12 and ptA13 of the pTNA extended structure (Supplementary Figure S3). This interaction suggests that Y594 could play an important role in coordinating the phosphodiester backbone during oligonucleotide synthesis, since it forms a direct contact to the DNA extended structure but only an indirect contact in the pTNA extended structure. Another novel interaction observed in the pTNA extended structure is a weak hydrogen bond between K592 and the O4′ atom on the sugar moiety of ptA13 (Figure 2D). Additionally, we noticed that the catalytic aspartate D542 was located further away from the terminal pTNA residue than its position in the all-DNA extended structure of Kod-RI, suggesting that the incoming ptNDP substrate may adopt a suboptimal geometry in the enzyme active site. The same observation was also made in the previously solved structure of Kod-RI containing a TNA extended primer (20). Collectively, these structural differences likely contribute to the reduced catalytic activity of pTNA synthesis by the Kod-RI TNA polymerase.

**Figure 3.**
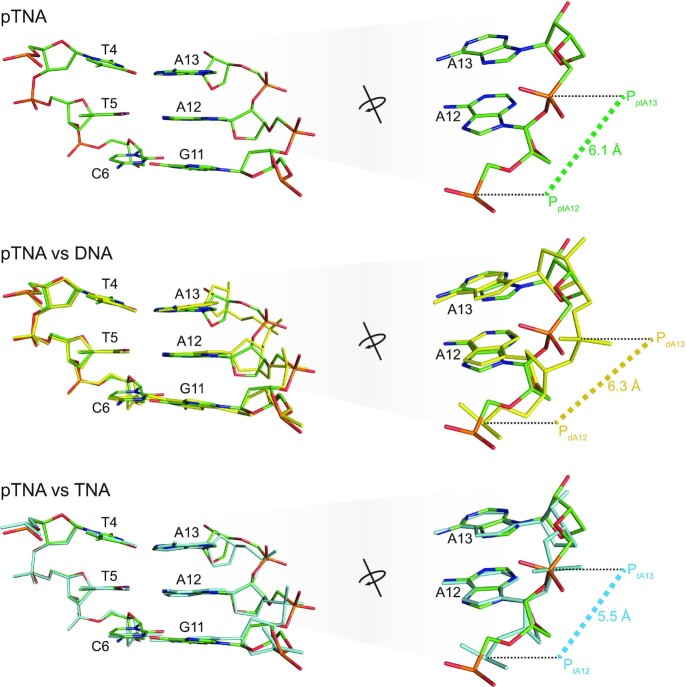
Active site view of the DNA, TNA and pTNA adducts. Structural comparison of the backbone moiety of the DNA (yellow), TNA (cyan) and pTNA (green) residues following primer extension with Kod-RI. Images on the right provide an elevated and rotated view of the overlay shown on the left in the Watson–Crick base pairing format with the templating strand. The P–P distances are provided in units of angstroms.

Alignment of the three crystal structures of Kod-RI enabled a direct comparison of the backbone conformation of the pTNA, DNA and TNA extended products. TNA exhibits a 5.5 Å P–P distance between adjacent phosphate groups (Figure 3), which is consistent with TNA having a five-atom backbone repeat unit rather than the more common six-atom repeat unit found in pTNA and DNA (39). By comparison, the analogous P–P distances in pTNA and DNA are 6.1 and 6.3 Å, respectively, which is consistent with a standard DNA duplex (40). Interestingly, pTNA adopts a backbone conformation that is strikingly similar to that of TNA, as evidenced from the overlays comparing pTNA to DNA and pTNA to TNA (Figure 3 and Supplementary Figure S4). In the pTNA–TNA overlay, the backbone residues occupy positions that are closer to the central axis of the primer–template duplex than the equivalent DNA extended product. Additionally, the phosphate group linking adenosine nucleotides 12 and 13 resides at the exact position for both the pTNA- and TNA-containing structures. By comparison, all of the atoms in the DNA backbone are positioned further away from the central axis with the phosphate group linking dA_12_ and dA_13_ shifted by ∼0.5 Å relative to its position in pTNA and TNA. The local base-pair and base-pair step parameters are reported in Supplementary Tables S4 and S5.

**Figure 4.**
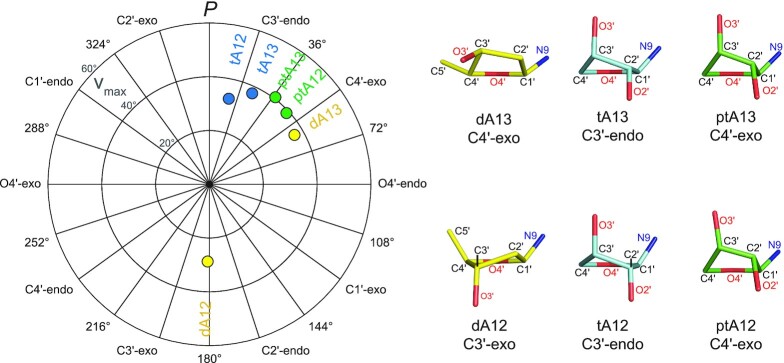
Pseudorotation wheel and sugar puckering. A pseudorotation wheel plotting the pseudorotation phase angle *P* (degrees) and the maximum sugar torsion angle *ν*_max_ (degrees) for the incorporated pTNA nucleotides (ptA12 and ptA13) and the analogous DNA (dA12 and dA13) and TNA nucleotides (tA12 and tA13). The puckered sugar moiety of each nucleotide is displayed in uniform orientation, where the C4′–O4′–C1′ atoms are positioned in the back.

We evaluated the torsion angles and pseudorotation parameters of the newly added adenosine residues in each of the three structures of Kod-RI (Figure 4, Supplementary Figure S5 and Supplementary Table S6). The sugar conformation of TNA has been reported to be in the range of C3′-*endo* to C4′-*exo* and is closer to C3′-*endo* in the active site of Kod-RI (*P*_tA12_ = 13° and *P*_tA13_ = 25°) (41). In contrast, the threose ring of pTNA adopts a sugar conformation that is C3′-*endo* (*P*_ptA13_ = 37°) and C4′-*exo* (*P*_ptA12_ = 47°) in the enzyme active site of Kod-RI. The extent of puckering versus planarity is described by *ν*_max_, which does not vary appreciably between the threose sugars of pTNA and TNA. Interestingly, the pTNA extended structure supports a computationally predicted model of a single 2′-deoxythreose phosphonate nucleotide that produced a sugar pucker in the range of the C3′-*endo* to C4′-*exo* conformations as investigated in the active site of HIV-1 reverse transcriptase (42). Similar analysis of the DNA extended structure reveals that the 2′-deoxyadenosine sugar at position 12 of the primer strand adopts a favorable C2′-*endo* conformation (*P*_dA12_ = 181°) that transitions to a C4′-*exo* (*P*_dA13_ = 60°) conformation at position 13 of the primer strand. This result is consistent with previous structure sugar conformations observed previously in crystal structures of DNA polymerases (43).

Analysis of the *χ* and *δ* torsion angle covariance (Supplementary Figure S6) indicates that the second DNA incorporation shifts to a lower *χ* value and exhibits a considerable 54° drop in the *δ* angle, moving toward an A-form helical geometry, while staying within the boundary of B-form structures. In contrast, the first pTNA and TNA nucleotides incorporated onto the 3′-end of the DNA primer (A_12_; *n* + 1) exhibit slightly higher *δ* values relative to the canonical B-form geometry of DNA and observe only a slight decrease in *χ* while maintaining their *δ* values for the second incorporation (A_13_; *n* + 2). In other reported polymerase structures, a similar conformational change for DNA nucleotides adopting a ‘northern’ A-form conformation has been reported with a higher crystallographic resolution for DNA nucleotides in Bst DNA polymerase (A-family, *Bacillus stearothermophilus*) incorporating DNA (43). Likewise, a structure of wild-type Kod bound to a DNA duplex without any incorporations shows the terminal primer nucleotide adopting a similar ‘northern’ A-from conformation (44). The observed changes in the sugar pucker of the DNA adducts may be necessary for positioning the 3′-terminal hydroxyl group for the subsequent nucleotide incorporation steps in the DNA polymerase cycle.

### Kod-RSGA mutations increase pTNA synthesis

**Figure 5.**
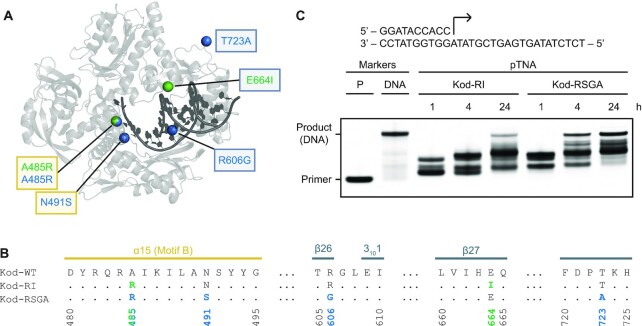
Engineered mutations in Kod-RSGA. (**A**) The overall architecture of Kod-RSGA TNA polymerase (gray) bound to a DNA duplex (black) is shown with the positions of the amino acid mutations observed in Kod-RI and Kod-RSGA highlighted as green and blue spheres, respectively. Mutations boxed in yellow and gray occur in the finger and thumb subdomains, respectively. (**B**) A sequence alignment highlights the secondary structures and positions of the engineered mutations. Both mutations in the finger are part of a conserved sequence known as motif B. (**C**) A time course demonstrates a faster synthesis rate for the Kod-RSGA. Both enzymes extended a 5′ IR680-labeled DNA primer annealed to a DNA template with ptNDP substrates. A DNA primer (11 nt) and a full-length DNA marker (18 nt) are provided. The reaction contained 0.5 μM IR800-labeled primer–template duplex, 100 μM ptNDPs and 0.5 μM Kod-RI or Kod-RSGA (pre-primed with 1 mM MnCl_2_), in 1× ThermoPol^®^ buffer for the 15-min intervals at 55°C.

To expand our findings with Kod-RI, we evaluated the ability of Kod-RSGA to direct the synthesis of pTNA on DNA templates. Kod-RSGA is a more efficient TNA polymerase that was recently discovered by programmed allelic mutagenesis (21). Similar to Kod-RI, Kod-RSGA contains the exonuclease silencing mutations (D141A and E143A) and shares the A485R gain-of-function mutation necessary for TNA synthesis. Additional mutations include N491S located in the finger subdomain, as well as the mutations R606G and T723A, both of which are located in the thumb subdomain (Figure 5A and B). Since Kod-RSGA has been reported to exhibit a 20-fold improvement in the rate of TNA synthesis relative to Kod-RI (21), we investigated the impact of these additional mutations on the rate of pTNA synthesis. Primer extension assays comparing Kod-RI and Kod-RSGA as polymerases for pTNA synthesis on a DNA template reveal that Kod-RSGA functions with higher overall activity than Kod-RI (Figure 5C). However, despite the enhanced activity of Kod-RSGA as a novel pTNA polymerase, this enzyme is still unable to generate full-length product. This is not an unexpected outcome, given that Kod-RSGA was originally evolved for TNA synthesis activity (21).

To better understand the activity of Kod-RSGA as a pTNA polymerase, we solved the X-ray crystal structure of Kod-RSGA in the post-catalytic state following two cycles of pTNA addition to the 3′-end of the DNA primer. The structure resolved to a resolution of 3.01 Å (Supplementary Table S3), enabling a detailed comparison with the equivalent structure of Kod-RI TNA polymerase. As expected, the Kod-RI/pTNA and Kod-RSGA/pTNA structures are highly homologous with a calculated Cα RMSD value of 0.561. The conformation of the pTNA nucleotides in Kod-RSGA strongly resembles the conformation of pTNA in Kod-RI in terms of torsion angles of the backbone and sugar pucker (Supplementary Table S6 and Supplementary Figure S7).

The biggest difference between the Kod-RI and Kod-RSGA structures extended with pTNA is the structural rearrangement observed as a result of the R606G mutation found in Kod-RSGA. As discussed previously, the R606 residue observed in Kod-RI forms a novel alternative conformation that leads to a strong ion pair interaction with the negatively charged phosphonate group connecting the newly added ptA12 and ptA13 nucleotides (Figure 2D). In Kod-RSGA, this position is mutated to a glycine residue, which allows Y594 to form a strong hydrogen bond to the same phosphate group previously contacted by the R606. This contact is predicted to be important for synthesis, as it is also observed in the structure of wild-type Kod DNA polymerase bound to an all-DNA substrate (44).

## DISCUSSION

Understanding the structural determinants that allow engineered polymerases to transcribe and reverse transcribe synthetic genetic polymers with orthogonal base pairing properties is an important step toward the creation of a genetic firewall that can separate the synthetic biology information produced by humanity from the natural biological information of the cell (15). Such endeavors will almost certainly be aided by the development of more efficient XNA polymerases, as the length constraints imposed by synthetic biology vastly exceed what is currently possible by solid-phase XNA synthesis. At a minimum, XNA polymerases will be needed to copy synthetic biology information from DNA into XNA, replicate XNA independent of DNA and reverse transcribe XNA back into DNA. Although we are still years away from replicating XNA polymers in a model cellular organism, the process of generating the enzymes required to achieve this goal will provide important new insights into the molecular recognition of XNA substrates by laboratory-evolved XNA polymerases.

Our study represents an important first step in the direction of understanding how engineered polymerases are able to copy genetic information from DNA into XNA when the XNA system being synthesized does not cross-pair with DNA. To our knowledge, all previous crystal structures of XNA polymerases describe enzymes that recognize XNA substrates that are known to exchange genetic information with DNA through the formation of stable antiparallel Watson–Crick duplex structures that can occur free in solution (20,45–47). However, pTNA, like GNA before it, is an example of orthogonal genetic polymers that do not base pair with DNA, but for reasons that are unclear are still able to transfer information from DNA into XNA, or vice versa, using engineered polymerases that can synthesize the complementary strand (17,18). How an engineered polymerase is able to recognize an artificial genetic polymer that does not form a stable duplex with the templating strand is an interesting question that could offer new insights into the mechanism of polymerase-mediated oligonucleotide synthesis. The current work focuses on the crystal structure of the post-translocated complex in which a DNA duplex has been extended by two pTNA residues. This intermediate in the pTNA synthesis pathway was viewed as a logical starting point, as similar complexes have previously yielded high-resolution structures (20).

Our structures reveal that pTNA bears a remarkable likeness to TNA when bound as a substrate to the active site of an engineered polymerase. TNA, a chemical analogue of pTNA, has been extensively studied as a substrate for polymerase recognition, both as a template and as a nucleoside triphosphate (19,21,48–50). Relative to TNA, which has a five-atom backbone repeat unit consisting of threofuranosyl nucleic acids connected by 3′→2′ phosphodiester linkages, pTNA comprises a six-atom backbone repeat unit in which a methyl group has been inserted between the 3′ hydroxyl group and the phosphorus atom (Figure 1). This chemical change transitions TNA from a synthetic genetic polymer that can base pair with DNA and RNA to one that cannot, implying that pTNA should be structurally different from TNA. However, despite this prediction, our crystal structures reveal that both XNA systems adopt similar sugar puckers with conformations in the C3′-*endo* and O4′-*endo* range and give rise to nucleic acid backbones with their phosphate groups located at identical positions. One possible interpretation of this observation is that the polymerase is forcing the substrates to adopt a specific conformation that is required for XNA synthesis and the threose sugars are adjusting to meet these constraints. Since it is difficult to answer this question with the current data, an important future goal will be to determine the structures of pTNA and TNA homoduplexes in the absence of a bound polymerase. A related future goal will be to solve crystal structures of the polymerase bound to longer pTNA extension products to see at which point the pTNA–DNA strands begin to dissociate from one another.

**Figure 6.**
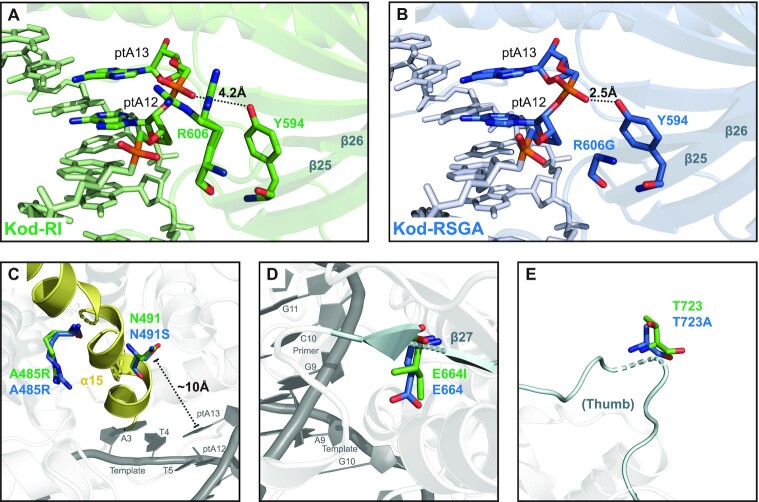
Structural consequences of the engineered mutations. Panels (**A**) and (**B**) show the active sites of Kod-RI and Kod-RSGA, respectively, highlighting the interactions of the pTNA backbone with residues 606 and Y594. Panels (**C**), (**D**) and (**E**) show an overlay of the engineered mutations in Kod-RI (green) and Kod-RSGA (blue). The α15 helix of the finger subdomain (yellow) and the β27 sheet and the loop containing position 723 in the thumb subdomain (cyan) are highlighted.

Although the backbone conformations of pTNA and TNA are structurally similar, they are distinct enough to form different interactions with the polymerase. These differences may be required in order to maintain Watson–Crick base pairing with the DNA template. The conformational transition observed for the 3′ terminal deoxyribose sugar in the control DNA structure is likely a paradigmatic example of how natural wild-type polymerases function as compared to the more rigid conformations observed for the threose sugars in the pTNA and TNA bound structures. Other notable differences include contacts to pTNA when the substrate is bound to Kod-RI versus Kod-RSGA. For example, the Kod-RSGA structure restores the hydrogen bonding interaction between Y594 and the phosphate group linking ptA12 to ptA13, which is also present in the DNA control structure and thought to be important for DNA synthesis (Figure 6). We postulate that this interaction may also be important for pTNA synthesis, since Kod-RSGA is a more efficient pTNA polymerase than Kod-RI.

Finally, it is interesting to note that the polymerase mutations previously known to be beneficial for TNA synthesis also appear to benefit pTNA synthesis. The growing body of information coming from polymerase engineering studies is helping to identify the structural determinants of XNA synthesis. As these studies continue, it will be interesting to see how known hot spots, such as V93, L408, A485, N491, I521 and E664, correlate to improved activity when additional gain-of-function mutations are discovered elsewhere in the polymerase. Are these mutations merely reducing the stringency of substrate recognition or are they driving the polymerase toward a new chemical function? As more structures are solved for XNA polymerases, and additional XNA polymerases are better characterized biochemically, it will be interesting to assess how easy or difficult it is for a natural DNA polymerase to erase its memory for DNA in favor of a synthetic genetic material that may bear little chemical resemblance to the genetic polymers of life. Indeed, pTNA with modifications in both the sugar and phosphate backbone could be an important step in this new direction.

## CONCLUSION

We describe the first crystal structures of an orthogonal genetic polymer bound to an engineered polymerase. Our results indicate that engineered polymerases recognize pTNA differently from TNA even though pTNA appears to be structurally similar to TNA when bound to the enzyme active site. These findings highlight the challenges of developing new XNA polymerases by directed evolution, as even structurally related substrates require unique intermolecular interactions in order to allow for efficient XNA synthesis on DNA templates.

## DATA AVAILABILITY

Atomic coordinates and structure factors for the reported crystal structures of Kod-RI/pTNA, Kod-RI/DNA and Kod-RSGA/pTNA have been deposited with the Protein Data bank under accession numbers 7RSR, 7RSS and 7TQW, respectively.

## Supplementary Material

gkac792_Supplemental_File

## Notes

Present address: Chao Liu, School of Pharmaceutical Sciences, Shandong University, Jinan 250012, China.
