# Supplementary material for: Crystallographic analysis of engineered polymerases synthesizing phosphonomethylthreosyl nucleic acid
Source: Nucleic Acids Res. 2022 Sep 17;50(17):9663–74. doi: 10.1093/nar/gkac792 (PMC9508818; doi:10.1093/nar/gkac792)
Supplement: gkac792_Supplemental_File [file gkac792_supplemental_file.pdf]

Supplementary Information

Crystallographic Analysis of Engineered Polymerases Synthesizing Phosphonomethylthreosyl Nucleic Acid

Mohammad Hajjar[1], Nicholas Chim[1], Chao Liu[2 ⊥], Piet Herdewijn[2],
and John Chaput[1,3,4,5,*]

[1] Department of Pharmaceutical Sciences, University of California, Irvine, CA 92697-3958, USA
[2] Medicinal Chemistry, Rega Institute for Medical Research, KU Leuven, Herestraat 49, 3000 Leuven
[3] Department of Chemistry, University of California, Irvine, CA 92697-3958, USA
[4] Department of Molecular Biology and Biochemistry, University of California, Irvine, CA 92697-3958, USA
[5] Department of Chemical and Biomolecular Engineering, University of California, Irvine, CA 92697-3958, USA
[⊥]Current address: School of Pharmaceutical Sciences, Shandong University, Jinan 250012, China.

Table of Contents

Table S1. Oligonucleotide Sequences

Table S2. Buffer Compositions

Table S3. Data collection and refinement statistics

Table S4. Local Base-Pair Parameters

Table S5. Local Base-Pair Step Parameters

Table S6. Torsion Angles and Pseudorotation Parameters

Figure S1. Kod Structural Overview

Figure S2. Kod-RI incorporating TNA and Active Site Overlays

Figure S3. Kod-RI/pTNA: Y594 Water Coordination

Figure S4. Backbone Comparison: Comprehensive Overlay

Figure S5. Torsion Angles Definition

Figure S6 Incorporated Nucleotides (χ,δ) Covariance

Figure S7. pTNA's Sugar Puckering in Kod-RSGA

Supplementary Table S1. Oligonucleotide Sequences

Name	Oligonucleotide sequence (5' – 3')	Comments
P_f	ATCCATATGATCCTCGACACTGACTAC	Cloning
P_r	ACGCATGCGGCCGCTCAAGTTCCCTTCGGCTTCAGCCA	
P_l	/5IRD/ GGATACCACC	Primer extensions
T_l	TCTCTATAGTGAGTCGTATAGGTGGTATCC	
P_c	CGCGAACTGCG	Crystal complex
T_c	/Cy5/ AAATTCGCAGTTCGCG	

Supplementary Table S2. Buffer Compositions

Name	Buffer Composition	Comments
Kod low salt/lysis buffer	10 mM Tris.Cl pH 7.5, 100 mM NaCl, 0.1 mM EDTA, 1 mM DTT, 10% glycerol, 0.125 mg mL^{-1} egg hen lysozyme when lysing cells	Purification
Kod high salt buffer	10 mM Tris.Cl pH 7.5, 1 M NaCl, 0.1 mM EDTA, 1 mM DTT, 10% glycerol	
Kod buffer	50 mM Tris.Cl pH 8.5, 200 mM NaCl, 0.1 mM EDTA, 1 mM DTT	Protein storage; Crystallization
Thermopol® (NEB)	20 mM Tris-HCl, 10 mM $(NH_4)_2SO_4$, 10 mM KCl, 2 mM $MgSO_4$, 0.1% Triton® X-100 pH 8.8	Primer extensions
Stop Buffer	95% formamide, 25 mM EDTA, pH 8.0	

Supplementary Table S3. Data Collection and Refinement Statistics. The statistics for the highest-resolution shell are shown in parentheses.

	**Kod-RI DNA**	**Kod-RI pTNA**	**Kod-RSGA pTNA**
**Data Collection**			
Space group	*P* 2 2_1 2_1	*P* 2 2_1 2_1	*P* 2 2_1 2_1
Cell Dimensions			
a, *b*, *c* (Å)	66.3, 112.0, 149.2	65.8, 111.6, 149.0	64.1, 111.5, 144.3
a, b, g (º)	90.0, 90.0, 90.0	90.0, 90.0, 90.0	90.0, 90.0, 90.0
Resolution (Å)	57.03-2.71 (2.81-2.71)	40.92-1.98 (2.05-1.98)	44.04-3.01 (3.12-3.01)
Total reflections	61392 (6062)	552026 (55933)	68272 (6983)
Unique reflections	30746 (3037)	77125 (7617)	19530 (1955)
Redundancy	2.0 (2.0)	7.2 (7.3)	3.5 (3.6)
Completeness (%)	99.36 (99.80)	99.90 (99.97)	91.96 (94.51)
$I / \sigma I$	13.14 (4.74)	17.86 (2.46)	10.33 (2.44)
Wilson B-factor	47.10	36.59	54.99
R_{merge}	0.026 (0.137)	0.074 (1.204)	0.1458 (0.7314)
CC1/2	0.998 (0.949)	0.999 (0.741)	0.991 (0.810)
CC*	1 (0.987)	1 (0.923)	0.998 (0.946)
**Refinement**			
Resolution (Å)	2.71	1.98	3.01
No. of reflections	30731 (3037)	77092 (7615)	19492 (1944)
R_{work}/R_{free}	0.161/0.226 (0.188/0.254)	0.176/0.212 (0.249/0.243)	0.176/0.270 (0.258/0.365)
No. atoms	6883	7052	6777
Protein/DNA	6798	6767	6735
ptA 12 & 13	-	42	42
Water	85	243	-
B-factors	52.35	52.71	54.24
Protein/DNA	52.47	52.91	54.22
ptA 12 & 13	N/A	34.71	55.84
Water	42.42	50.37	-
Clashscores	5.38	4.48	12.09
RMS dev. (Bonds; Å)	0.008	0.008	0.010
RMS dev. (Angles; º)	1.22	1.16	1.32
**PDB ID**	7RSS	7RSR	7TQW

Supplementary Table S4. Local Base-Pair Parameters.

BASE PAIR	Shear (Å)	Stretch (Å)	Stagger (Å)	Buckle (°)	Propeller (°)	Opening (°)
**RI/DNA**						
dA13 - dT4	-0.21	-0.12	0.35	-24.29	-10.10	8.20
dA12 - dT5	0.20	-0.19	-0.20	-3.79	-2.24	0.36
dG11 - dC6	0.12	-0.21	0.16	-1.57	-13.73	4.38
**RI/TNA**						
tA13 - dT4	-0.25	-0.37	0.29	-16.69	-15.14	1.70
tA12 - dT5	-0.30	-0.20	-0.32	-4.28	-4.75	1.76
dG11 - dC6	0.37	-0.25	-0.22	-5.48	-4.14	1.47
**RI/pTNA**						
ptA13 - dT4	-0.15	-0.20	0.24	-11.36	-15.97	3.96
ptA12 - dT5	-0.04	-0.15	-0.38	2.77	-3.40	-1.06
dG11 - dC6	0.31	-0.21	0.34	-10.99	-13.44	3.51
**RSGA/pTNA**						
ptA13 - dT4	-0.19	-0.15	0.29	-16.7	-25.13	1.18
ptA12 - dT5	-0.44	-0.10	0.42	-8.42	-13.20	-6.01
dG11 - dC6	0.55	-0.15	-0.01	-9.00	-0.43	4.82

Supplementary Table S5. Local Base-Pair Step Parameters.

STEP	Shift (Å)	Slide (Å)	Rise (Å)	Tilt (°)	Roll (°)	Twist (°)
**RI/DNA**						
dT4 dT5 / dA12 dA13	-0.50	-0.08	2.83	5.82	8.19	29.75
dT5 dC6 / dG11 dA12	0.66	-0.55	3.31	-4.09	7.12	40.23
**RI/TNA**						
dT4 dT5 / tA12 tA13	-0.29	-0.41	3.02	6.36	5.35	28.08
dT5 dC6 / dG11 tA12	0.41	-0.29	3.39	0.33	0.77	43.30
**RI/pTNA**						
dT4 dT5 / ptA12 ptA13	-0.19	-0.30	2.97	6.93	6.15	31.35
dT5 dC6 / dG11 ptA12	0.82	-0.69	3.64	-4.63	0.07	42.36
**RSGA/pTNA**						
dT4 dT5 / ptA12 ptA13	-0.70	-0.48	3.16	-0.28	10.07	30.50
dT5 dC6 / dG11 ptA12	1.04	-0.57	3.47	-0.37	-9.14	44.77

Supplementary Table S6. Torsion Angles and Pseudorotation Parameters.

	α	β	γ	δ	ε	ζ	χ	ν_0	ν_1	ν_2	ν_3	ν_4	ν_{max}	P
**RI/DNA**														
dA13	-77.7	167.4	51.5	91.2	N/A	N/A	-121.7	-24.9	2.9	18.4	-33.1	36.5	36.6	59.9
dA12	-71.7	-151.6	42.0	145.0	-167.5	-92.3	-103.5	-8.9	23.6	-28.8	24.5	-10.0	28.8	181.3
dG11	84.1	-118.6	-160.3	94.2	-150.5	-67.4	-172.3	-9.3	-7.7	20.7	-26.5	22.4	26.4	38.5
**RI/TNA**														
tA13	-88.9	N/A	168.3	159.0	N/A	N/A	-101.1	-5.4	-17.7	33.8	-36.6	24.7	37.3	25.3
tA12	-126.5	N/A	-135	161.4	-108.4	-83.2	-85.3	2.6	-21.3	31.88	-30.0	16.1	32.6	12.8
dG11	-56.6	161.9	49.0	130.1	177.1	-88.8	-126.3	-35.5	40.7	-30.3	10.9	15.1	40.0	139.3
**RI/pTNA**														
ptA13	66.0	-149.9	176.4	162.0	N/A	N/A	-119.1	-14.4	-12.7	32.3	-41.1	32.6	40.5	37.2
ptA12	-39.2	-147.4	-63.4	163.2	-84.6	-160.3	-92.7	-20.0	-4.9	26.5	-37.8	35.1	39.0	47.2
dG11	86.0	-124.8	-157.0	113.6	-134.6	-75.3	-166.7	-22.4	17.8	-7.0	-5.8	17.7	21.8	108.6
**RSGA/pTNA**														
ptA13	68.9	-160.1	-177.8	166.8	N/A	N/A	-107.2	-17.3	-11.7	34.0	-44.6	36.1	44.1	39.5
ptA12	-36.2	-148.4	-68.9	145.5	-93.3	-149.2	-78.2	-11.3	-6.4	20.3	-27.1	22.7	27.0	41.2
dG11	95.1	-125.6	-170.5	129.9	-135.5	-87.3	-154.9	-26.8	32.9	-26.3	11.7	9.4	32.2	144.7

The torsion angles are reported in degrees for the two incorporated adenosine nucleotides, A12 and A13, and the preceding nucleotide of the primer, dG11. A pictorial definition of the torsion angles is provided in SI Fig 2. The angles α, β, γ, δ, ε, and ζ are assigned to the exocyclic backbone of the genetic polymers. The β angle is omitted for TNA. The ε and ζ angles non-computable for the terminal A13 nucleotides. The χ angle is torsion angle about the glycosidic bond. The angles ν 0 through 4 describe the sugar moiety's endocyclic torsion angles. The ν_{max} is the maximum torsion angle and is reflective of the degree of puckering; a ν_{max} of 0° reflects a planar furanose ring. The pseudorotation phase angle, P, ranges from 0° to 360° and $P = 0°$ is defined such that the torsion angle ν_2 is maximally positive.

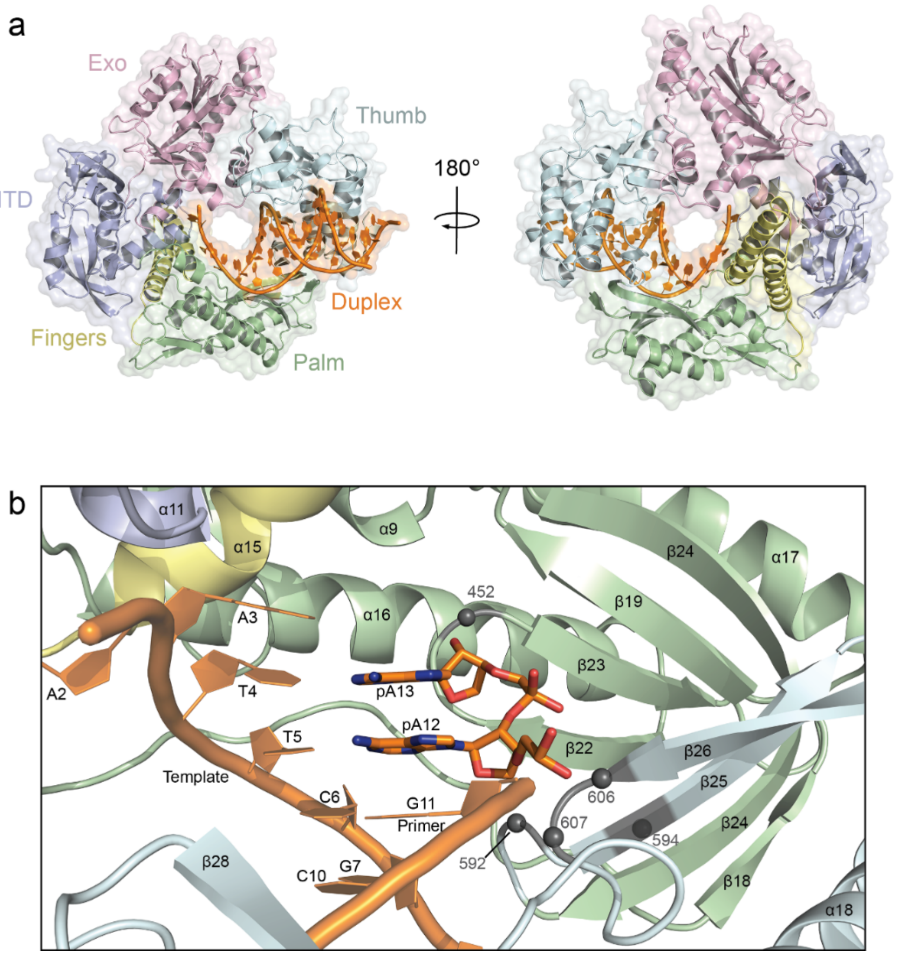

Supplementary Figure S1. Kod Structural Overview (a) The global architecture of Kod bound to a primer-template duplex. The front and back view showing a surface rendering overlayed on a cartoon representation is colored by protein subdomain. The primer-template duplex is shown in orange. (b) A view of the active site is shown with the exonuclease domain hidden to avoid view obstruction. Positions with residues that interact with the incorporated pTNA are shown as gray spheres.

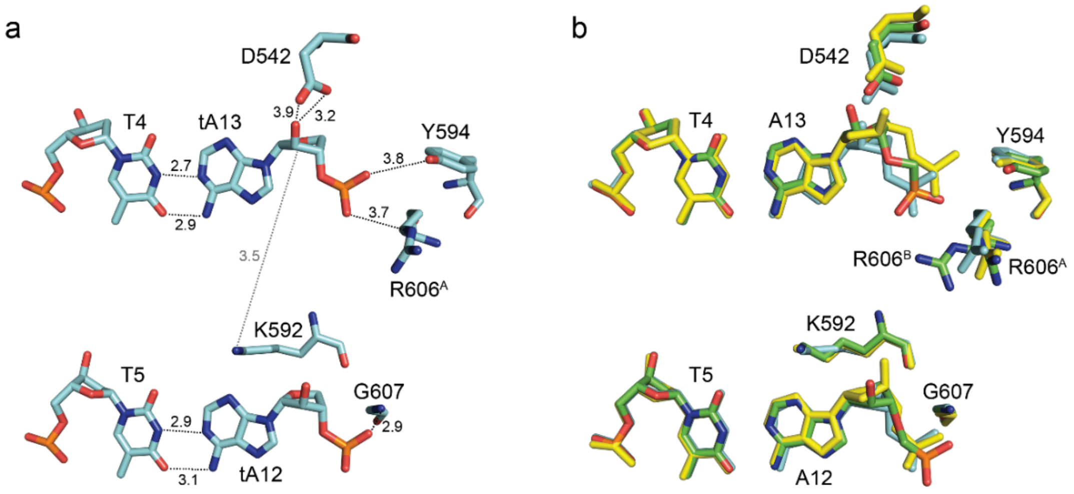

Supplementary Figure S2. Kod-RI Incorporating TNA and Active Site Overlays. (a) A panel equivalent to main text Figure 2b-d for TNA incorporations showing base pairing and protein interactions in angstroms. (b) An overlay of the incorporated DNA, TNA, pTNA is shown, highlighting the backbone variation in context of the active site.

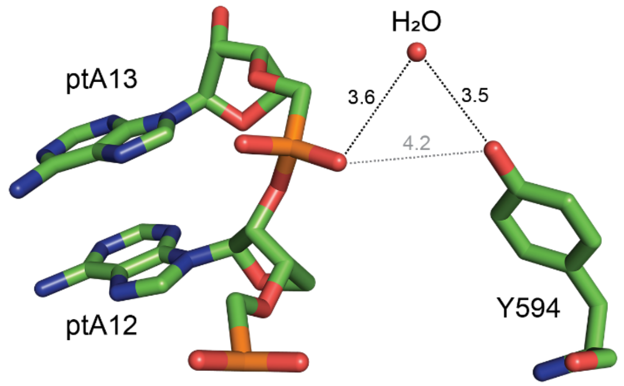

Supplementary Figure S3. Tyrosine 594 Forms a Bridging Water Interaction. In the structure of Kod-RI incorporating pTNA, tyrosine 594 is 4.2 Å away from the phosphonate backbone. Instead of hydrogen bonding, compensatory water coordination is observed.

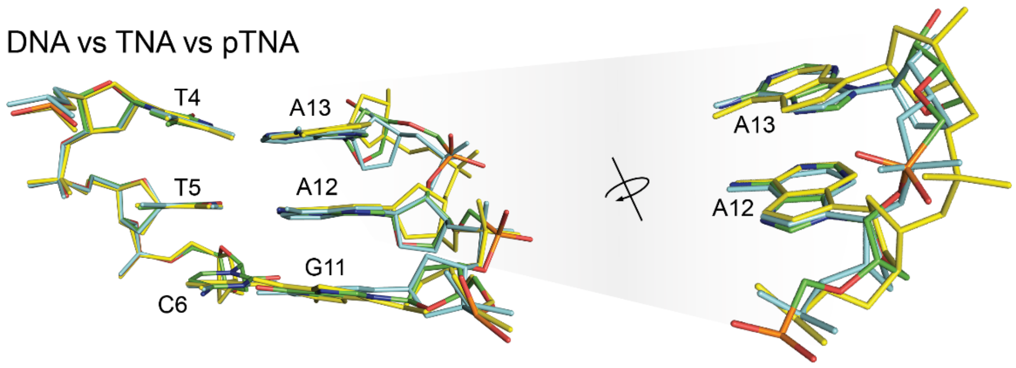

Supplementary Figure 4. A Backbone Comparison of Newly Added Nucleotides . A view identical to that of Figure 3, overlaying RI/pTNA (green, colored by element), RI/DNA (solid yellow), and RI/TNA (solid cyan) at once.

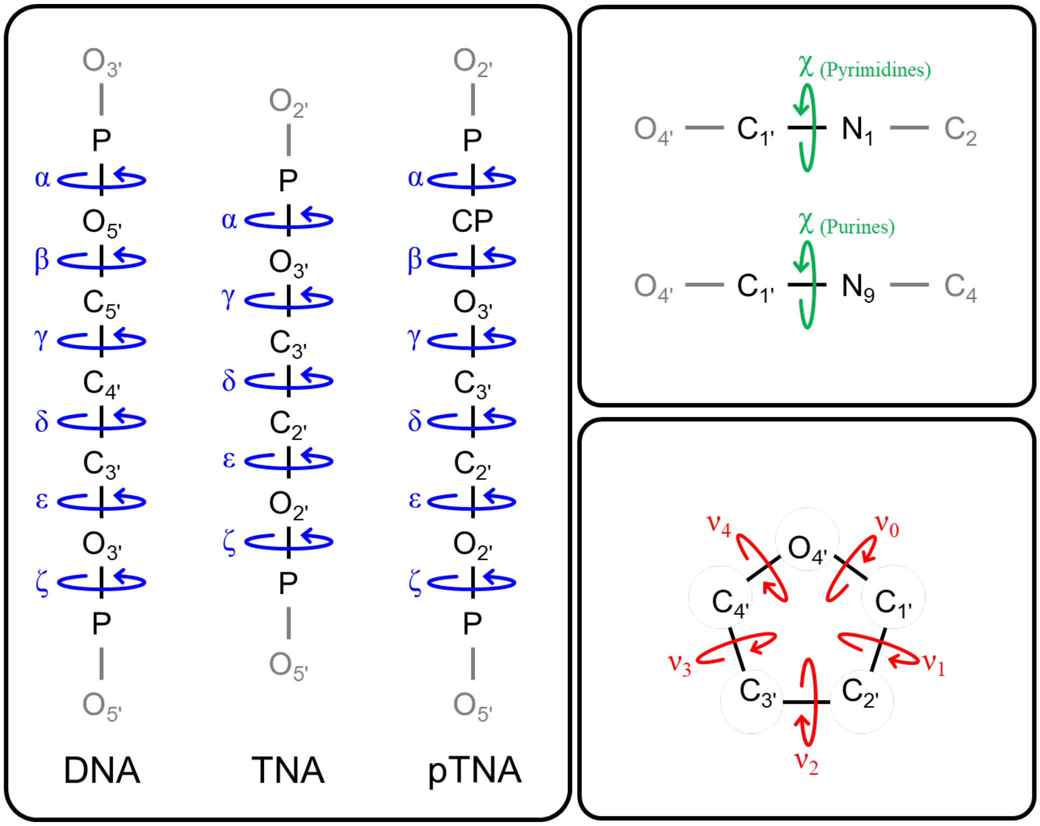

Supplementary Figure 5. Torsion Angles Definition. A torsion angle about a bond is defined by 4 atoms, the two bonding atoms, and an additional atom bonded to each. Pictorial definitions of the exocyclic backbone torsion angles in the backbones of DNA, TNA, and pTNA are shown in blue. TNA's shortened backbone lacks the β angle. For all genetic systems, the torsion angles about the glycosidic bond for pyrimidines and purines are defined in green, and the sugar endocyclic torsion angles are defined in red.

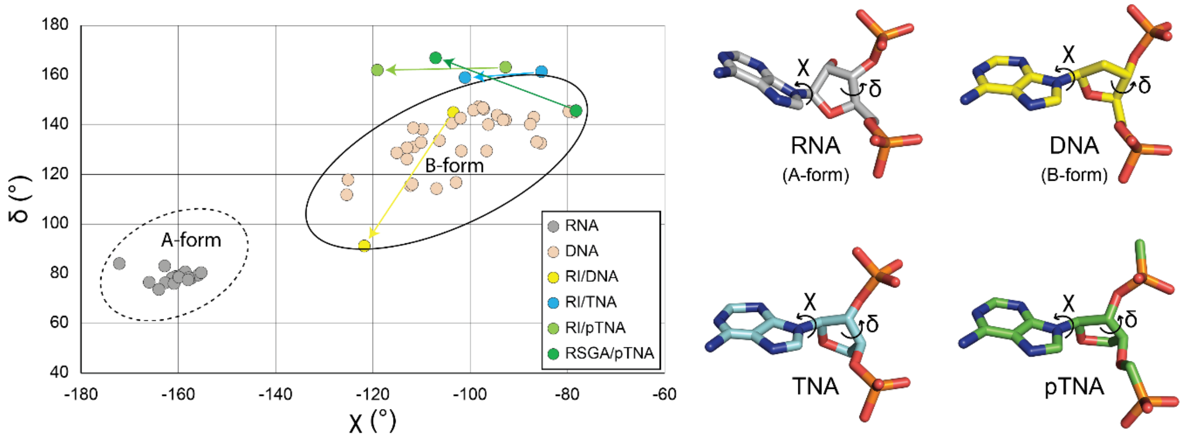

Supplementary Figure 6. Incorporated Nucleotides (χ,δ) Covariance. The (χ,δ) covariance is used to analyze the helical geometry of the TNA and pTNA nucleotides with canonical A-form RNA (PDB ID: 3ND4) and B-form DNA (PDB ID: 3BSE) nucleotides as a reference. Arrows drawn on the plot represent the shift in (χ,δ) covariance between the first (A12; n+1) and the second incorporations (A13; n+2). On the right, representative monomers, that is, A12 for DNA, TNA, and pTNA, are shown in comparison to a canonical A-form RNA monomer.

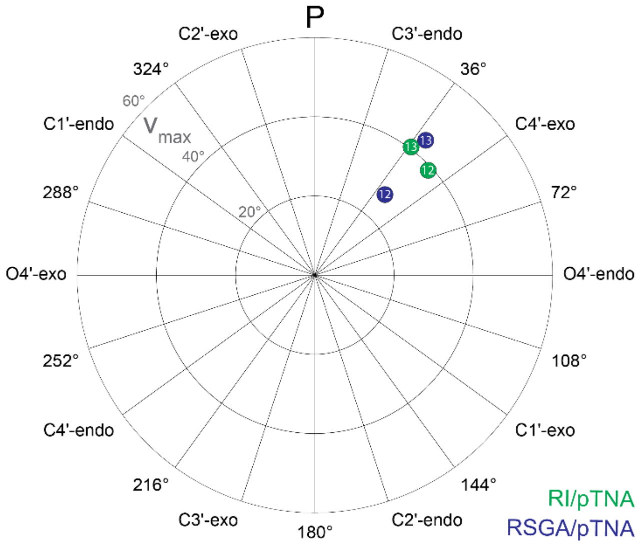

Supplementary Figure 7. pTNA's Sugar Puckering in Kod-RSGA. The pseudorotation parameters of pTNA in RI/pTNA (green) and RSGA/pTNA (blue) are plotted, depicting the slight increase in the extent of puckering (ν_{max}).
